# Epistatic interactions between sex chromosomes and autosomes can affect the stability of sex determination systems

**DOI:** 10.1111/jeb.13939

**Published:** 2021-10-01

**Authors:** Martijn A. Schenkel, Leo W. Beukeboom, Ido Pen

**Affiliations:** ^1^ Groningen Institute for Evolutionary Life Sciences University of Groningen Groningen The Netherlands

**Keywords:** epistasis, female heterogamety, male heterogamety, sex determination transitions, sexual selection & conflicts

## Abstract

Sex determination (SD) is an essential and ancient developmental process, but the genetic systems that regulate this process are surprisingly variable. Why SD mechanisms vary so much is a longstanding question in evolutionary biology. SD genes are generally located on sex chromosomes which also carry genes that interact epistatically with autosomes to affect fitness. How this affects the evolutionary stability of SD mechanisms is still unknown. Here, we explore how epistatic interactions between a sexually antagonistic (SA) non‐SD gene, located on either an ancestral or novel sex chromosome, and an autosomal gene affect the conditions under which an evolutionary transition to a new SD system occurs. We find that when the SD gene is linked to an ancestral sex‐chromosomal gene which engages in epistatic interactions, epistasis enhances the stability of the sex chromosomes so that they are retained under conditions where transitions would otherwise occur. This occurs both when weaker fitness effects are associated with the ancestral sex chromosome pair or stronger fitness effects associated with a newly evolved SD gene. However, the probability that novel SD genes spread is unaffected if they arise near genes involved in epistasis. This discrepancy occurs because, on autosomes, SA allele frequencies are typically lower than on sex chromosomes. In our model, increased frequencies of these alleles contribute to a higher frequency of epistasis which may therefore more readily occur on sex chromosomes. Because sex chromosome–autosome interactions are abundant and can take several forms, they may play a large role in maintaining sex chromosomes.

## Graphical abstract

Epistatic interactions between sex chromosomes and autosomes can cause increased stability of the sex determination system, favouring its maintenance over the invasion of novel systems, or decreased stability where an invasion of a novel sex determination system becomes favoured. The nature of these effects on the stability of sex determination system depends strongly on the type of epistatic interaction, its strength, and the type of newly‐invading sex determination gene.

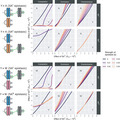

## 1 INTRODUCTION

In sexually reproducing species, the process of sex determination (SD) is an essential part of an individual's development, but the manner in which the sexual phenotype is set is far from conserved. An astounding variety of SD mechanisms has been described (Bachtrog et al., 2014; Beukeboom & Perrin, 2014); among organisms with genetic sex determination systems (GSD), there exists large variation in the genes and mechanisms that control the sexual phenotype. In most GSD systems, the primary SD gene lies on a sex chromosome, resulting in either male heterogamety (males XY, females XX) or female heterogamety (females ZW, males ZZ). In some organismal groups, the SD gene (and by extension, the sex chromosome pair) that determines sex is strongly conserved, such as the *SRY* gene and the XY system of therian mammals (Graves, 2006). However, other organismal groups exhibit substantially more variation, with different sex chromosome systems present in different species (Vicoso, 2019), such as in lizards (Ezaz et al., 2009; Pokorná & Kratochvíl, 2016), teleost fishes (Mank, 2009) and flies (Vicoso & Bachtrog, 2015). In addition to interspecific variation in SD mechanisms, intraspecific SD variation exists in several species, such as the southern platyfish *Xiphophorus maculatus*, in which X‐, Y‐ and W‐chromosomes are found (Orzack et al., 1980), and the housefly *Musca domestica*, in which some populations have an XY system and others a ZW system (Feldmeyer et al., 2008; Hamm et al., 2015). The variability of SD mechanisms between and within organismal groups suggests that evolutionary turnovers between SD systems occur readily (Meisel, 2020; Vicoso, 2019).

Various population genetic models have been developed for evolutionary turnovers in SD systems (reviewed in van Doorn, 2014), of which two are of most interest here. First, sex ratio selection can favour a new SD gene when it induces development into the sex with the higher fitness, typically the minority sex (Fisher, 1930; Wilkins, 1995, but see Pen, 2006). Sex ratios can be biased due to, for example, sex chromosome meiotic drive (Jaenike, 2001; Kozielska et al., 2010), and selection can then favour a new SD gene that brings the sex ratio closer to 50:50. However, sex ratio selection can also favour rather than counteract deviations from equal sex ratios (Uller et al., 2007), and SD genes may also evolve when they actually cause such deviations (Kuijper & Pen, 2014). Second, linkage with sexually antagonistic (SA) loci has been proposed as a selective force in SD turnovers. As the regions flanking an SD locus are transmitted through males and females at different rates, SA loci can become genetically differentiated between the sexes. For example, a male‐determining allele might become linked to a male‐beneficial allele (on a primordial Y‐chromosome) whereas chromosomes lacking the male‐determining allele can become enriched for female‐beneficial alleles (X‐chromosome) (Charlesworth et al., 2014; Jordan & Charlesworth, 2012; Rice, 1984). Effectively, the SA locus and the SD locus evolve to form a co‐adapted gene complex, and depending on the fitness effects and degree of linkage of SA and SD loci, the new gene complex may spread (van Doorn & Kirkpatrick, 2007, 2010).

The acquisition of an SD gene on a chromosome initiates a process of sex chromosome differentiation (reviewed in Bachtrog et al., 2011; Charlesworth et al., 2005; Schenkel & Beukeboom, 2016). SA genes are expected to accumulate on the sex chromosomes along with the evolution of suppressed recombination on the Y‐chromosome (or the W‐chromosome in ZW systems) (Rice, 1987, 1996). Subsequent degradation and masculinization of the Y‐chromosome can help stabilize the SD system, by preventing it from becoming either fixed or lost (Marin & Baker, 1998). Overall, the stability of an SD mechanism can be affected by the association between the SD gene and nearby linked genes, and depending on the function of these linked genes different selective pressures may act on the SD gene.

Models on the evolution of SD mechanisms often focus on direct selection on the SD gene or the sex chromosome on which it is located. However, sex chromosomes represent only a fraction of the genome and the autosomes typically make up the majority. Besides direct effects on the individual (e.g., by determining its sex), sex chromosomes may also have indirect effects through interactions with other (autosomal) genes, such as in humans (Bellott et al., 2014) and *Drosophila melanogaster* (Jiang et al., 2010; Lemos et al., 2008); in both species, the Y‐chromosome harbours multiple genes that extensively regulate X‐chromosomal and/or autosomal gene expression, and thereby eventually affect fitness. The evolution of gene expression differences and dosage compensation in recently formed sex chromosome systems suggests that even from an early point on sex chromosomes may interact with autosomes to affect fitness (Archer et al., 2017; Lachance et al., 2011; Zhou & Bachtrog, 2012). This is not surprising as sex chromosomes are thought to originate from autosomes (Ohno, 1967) and may prior to becoming sex chromosomes have been involved in autosome–autosome epistatic interactions. Although SA genes may accumulate on the sex chromosomes, they could also remain on the autosomes but become regulated by sex‐chromosomal genes that control their expression (Parsch & Ellegren, 2013). Thus, although the sex chromosomes represent a specialized part of the genome, they can have crucial effects on autosomal gene expression and individual fitness by interacting with other components of the genome. Whether and how these interactions can influence the stability of SD mechanisms have however not been investigated yet.

Our aim is to investigate whether epistasis between autosomes and sex chromosomes can affect the stability of SD systems. We build on previous work by Van Doorn and Kirkpatrick (2007, 2010) who investigated the influence of SA loci on transitions between SD mechanisms. Their models focus on two unlinked SD genes, each of which is linked to an SA locus. This mimics a situation in which the ancestral sex chromosome pair has begun differentiating into a full‐fledged sex chromosome as described above, but has not yet undergone extensive genetic differentiation; the novel SD gene then arises near an autosomal SA locus. Depending on the selective pressures acting on the SA loci, the new SD gene may then invade or not. Such transitions can be between identical sex chromosome systems (e.g., between different male heterogamety systems; van Doorn & Kirkpatrick, 2007) or between different types of sex chromosome systems (e.g., male heterogamety to female heterogamety or vice versa; van Doorn & Kirkpatrick, 2010). We focus here specifically on how epistasis alters the scope for turnover as predicted by these previous models. Thus, we investigate how epistatic interactions can affect the occurrence of SD transitions.

## 2 METHODS

### 2.1 Model overview

**FIGURE 1:**
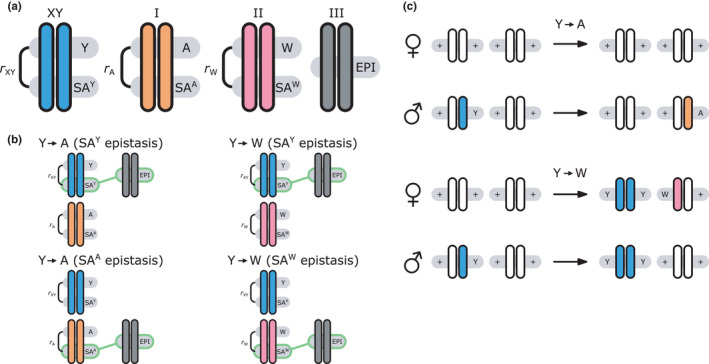
Model overview. (a) Genetic components of the model. All loci are labelled with their focal allele. Recombination rates between the SD and SA loci are given by $r_{\mathrm{XY}}$, $r_{A}$ and $r_{W}$ for linkage groups XY, I^A^ and II^W^, respectively. (b) SD transitions scenarios considered. Epistatic interactions between loci are indicated in green. Only linkage groups which harbour SD genes involved in the transition and the linkage group carrying the EPI locus are depicted. All scenarios start out with a population where Y is the ancestral SD locus into which we introduce a new SD allele (either A or W). (c) Male and female karyotypes before (left) and after (right) transitions. Coloured chromosomes indicate the presence of an SD gene (Y, A or W) whereas white chromosomes indicate absence of an SD gene

We provide here a conceptual description of our model; a more technical treatment is presented in the Appendix. We work with discrete, non‐overlapping generations and random mating in a population with an infinite size. Offspring genotypes are determined based on Mendelian segregation whilst accounting for recombination, followed by viability selection based on their relative fitness. Our model features a diploid genome consisting of four different linkage groups (Figure 1a). The first three linkage groups (XY, I^A^ and II^W^) each carry one SD locus and one SA locus that recombine at a rate r that can vary per linkage group. The fourth linkage group carries a single locus, called EPI, that interacts epistatically with the SA locus on XY, I^A^ or II^W^ to affect male fitness. Each locus has two possible alleles (referred to as the non‐focal and focal alleles). The non‐focal allele corresponds to a recessive allele without phenotypic effects (generally denoted +), whereas the focal allele affects the sex (for SD loci) or fitness (for SA loci) of an individual. We refer to the focal alleles by the name of their respective loci; all allele frequencies reported represent the frequencies of these focal alleles.

For the SD loci on XY and I^A^, the focal allele constitutes a male‐determining factor (Y and A, respectively), whereas the SD locus on II^W^ corresponds to a dominant female determiner (W) which overrides the action of Y. The terms male and female are interchangeable, and hence, the model also applies to, for example, competing female‐determining genes or transitions from female to male heterogamety. The SD genotypes that can be formed in either Y→A and Y→W transitions and their corresponding sex are listed in Table 1.

**TABLE 1:** Possible genotype combinations for SD loci and the resulting sex of the individual. Under Y→A and Y→W, the genotypes that can exist in each SD transition scenario are depicted

XY	I^A^	II^W^ ^a^	Sex	Y→A	Y→W
+/+	+/+	+/+	Female	✓	✓
Y/+	+/+	+/+	Male	✓	✓
Y/ Y	+/+	+/+	Male		✓
+/+	A/+	+/+	Male	✓	
Y/+	A/+	+/+	Male	✓^b^	
+/+	+/+	W/+	Female		✓
Y/+	+/+	W/+	Female		✓
Y/ Y	+/+	W/+	Female		✓

^a^ The W/W genotype at II^W^ cannot be obtained in our model as the W allele cannot be transmitted through males.

^b^ A low frequency of A alleles is introduced by mutation across all genotypes present in the population at that time; this results in small numbers of Y/+; A/+ individuals that decrease in frequency over time due to producing a 75% sex ratio (compared with favoured 50% sex ratios for males with a single Y or a single A allele).

Genotypic fitness is defined as the relative viability of individuals carrying a particular genotype. An individual's fitness is determined by the genotype at the SA loci, whose effects depend on the individual's sex. Fitness effects of the focal allele at a single SA locus are determined by its fitness effect sizes in males and females $s_{M}$ and $s_{F}$ in homozygotes and additionally the sex‐specific dominances for these effects in heterozygotes $h_{M}$ and $h_{F}$ (for details see Table 2). SA^Y^ and SA^A^ both have positive effects in males ($s_{M}>0$) and inversely negative effects in females ($s_{F}<0$). Conversely SA^W^ has positive effects in females but negative effects in males (See Table S1). A female's total fitness is given by the product of the fitness scores of all SA loci, that is:

(1a) $$w_{F}\;\;=w_{{\mathrm{SA}}^{Y}}\times w_{{\mathrm{SA}}^{A}}\times w_{{\mathrm{SA}}^{W}}$$

**TABLE 2:** Genotype by sex fitness effects for SA loci. A + is used to denote a wild‐type allele, and SA a focal allele (SA^Y^, SA^A^ or SA^W^). Each SA locus has sex‐specific dominance parameters ($h_{M}$ and $h_{F}$) and fitness parameters ($s_{M}$ and $s_{F}$). $s_{M}$×$s_{F}$<0 reflects SA selection at each locus. Default parameter values are used for $h_{M}=0.6,\;h_{F}=0.4$ for SA^Y^ and SA^A^ and $h_{M}=0.4,\;h_{F}=0.6$ for SA^W^

Sex	+/+	SA/+	SA/SA
Males	1	$1+h_{M}\times s_{M}$	$1+s_{M}$
Females	1	$1+h_{F}\times s_{F}$	$1+s_{F}$

Here, $w_{{\mathrm{SA}}^{Y}}$, $w_{{\mathrm{SA}}^{A}}$ and $w_{{\mathrm{SA}}^{W}}$ refer to the locus‐specific fitness scores at the SA^Y^, SA^A^ and SA^W^ loci. In males, epistasis can further affect fitness, and therefore their fitness is given by:

(1b) $$w_{M}=w_{{\mathrm{SA}}^{Y}}\times w_{{\mathrm{SA}}^{A}}\times w_{{\mathrm{SA}}^{W}}\times w_{\mathrm{EPI}}$$

Here $w_{\mathrm{EPI}}$ represents the fitness effect of epistasis. This is the outcome of interactions between an SA locus and the EPI locus.

### 2.2 Epistasis scenarios and epistatic fitness effects

We let EPI interact with different SA genes to reflect situations in which either the established SD gene or the novel invading SD gene is linked to an SA locus that interacts epistatically with an autosomal gene. Although we are not aware of specific examples in which SA loci are indeed involved in epistasis, the existence of such loci is highly likely given that in several species, sex chromosomes are both enriched for sexually antagonistic genetic variation and for genes that play important roles in regulating autosomal gene expression (e.g., Innocenti & Morrow, 2010; Lemos et al., 2008). Alternatively, if such functions are performed by independent but linked genes, these may segregate as a single supergene to the same effect. This scenario may be particularly relevant for sex chromosomes in which recombination suppression has recently begun to evolve (Rice, 1987). Epistatic interactions between EPI and an SA locus only occur in males, and their effects depend both on the genotype at the SA locus and the genotype at the EPI locus. We do not incorporate epistatic effects in females to limit the complexity of the model, whereas including it would likely only affect the dynamics of the model marginally. This is because the frequency of the focal allele at the SA^Y^ locus is reduced in females and hence epistasis involving this locus would already occur at very low rates in females. For SA^A^ and SA^W^, the focal alleles may be present at higher frequencies in females than SA^Y^. However, these loci are still autosomal prior to the SD transition, and therefore, the frequency of their focal alleles may only be slightly higher than the focal SA^Y^ allele, so that epistasis involving these loci is similarly rare. Nonetheless, when the assumption that epistasis is sex‐specific is not met, the scope for turnover from Y to A or W may differ slightly from that predicted by our model. Epistasis in general represents a situation in which the effect of one gene is modulated by another gene, and the manner in which such gene–gene interactions influence fitness may be modelled via numerous different approaches (reviewed in Wade et al., 2001). To explore all possibilities is therefore infeasible, and instead, we consider three standardized scenarios which we refer to as dominance, overdominance and coadaptation (see also Table 3). In effect, these epistasis types mimic different selective scenarios; directional selection for dominance epistasis (favouring increased frequencies for both the SA allele and EPI), stabilizing selection for overdominance epistasis (favouring SA/+; EPI/+ double heterozygotes) and disruptive selection for coadaptation epistasis (favouring either SA/SA; EPI/EPI or +/+; +/+ double homozygotes). Although numerous alternative epistasis types are conceivable, they ultimately conform to minor variations to those considered here in that they share an underlying selective scenario. Interactions between the SA locus and EPI affect male fitness multiplicatively according to the factor $w_{\mathrm{EPI}}=1+\sigma \varepsilon$, where ε denotes the epistasis effect size, and the binary factor σ determines whether or not epistasis occurs or not. Table 3 lists the values of σ for every genotype combination in the different epistasis scenarios.

**TABLE 3:** Epistatic interactions for different scenarios. The different numerical values indicate the factor σ in the epistasis term 1+σϵ, which determines the epistasis interaction effect

	EPI genotype	SA genotype		
		+/+	SA/+	SA/SA
Dominance	+/+	0	0	0
	EPI/+	0	1	1
	EPI/EPI	0	1	1
Overdominance	+/+	0	0	0
	EPI/+	0	1	0
	EPI/EPI	0	0	0
Coadaptation	+/+	1	0	0
	EPI/+	0	0	0
	EPI/EPI	0	0	1

### 2.3 Model initialization and sex determination transition types

In each scenario, we start with a standard XY system with a single male‐determining allele Y which is fixed on the paternally inherited copy in males. New SD genes are not present in the ancestral population but arise later by mutation. For each separate simulation, we randomly sample the parameter values associated with the fitness effects of each SA gene and likewise the epistasis effect size. All these parameter values are sampled from a uniform distribution with range (0, 0.05). In addition, we perform a set of simulations where the effect of epistasis is set to zero (ε=0) to validate that when epistasis has no effect, the type of epistasis does not affect the outcome of potential SD transitions (for a detailed explanation, see ‘Data analysis’). Parameter values are resampled for every new simulation so that each features a unique set of parameter values. For simplicity, we assume $s_{M}=‐s_{F}$ for each SA locus. The SA alleles considered in the simulation and the EPI allele have an initial frequency of 0.25 in both sexes and on both the maternal and the paternal chromosome. Given that the selective effects of the SA alleles and the epistasis effects are allowed to vary, the allele frequencies upon initialization are unlikely to correspond to an equilibrium state. We therefore include a burn‐in period of 10,000 generations during which the allele frequencies at the SA and EPI loci can evolve to an approximate equilibrium state. After this, the novel SD gene (A or W) is introduced at a low frequency (10^−4^). We continue the simulation until a total of 200,000 generations have been reached and determine whether an SD turnover took place by analysing the SD allele frequencies.

We consider here both transitions between different male heterogamety systems (Y replaced by A) and a transition from male to female heterogamety (Y fixed and W invades as a dominant female determiner) (Figure 1c). Because we also vary the SA gene interacting with EPI, this results in a total of four different SD transition scenarios, being (1) Y→A (SA^Y^ epistasis); (2) Y→A (SA^A^ epistasis); (3) Y→W (SA^Y^ epistasis); and (4) Y→W (SA^W^ epistasis) (Figure 1b). We focussed on the fitness effects of SA genes and the epistasis effect, and standardized other parameters such as recombination rates. These standardized parameter values for all transition scenarios are listed in Table S1. The selective effect parameters for the SA loci linked to SD loci involved (SA^Y^ and SA^A^ for the Y→A transition; SA^Y^ and SA^W^ for the Y→W transition) and the epistasis effect size ε were randomly sampled from uniform distributions with range (0, 0.05) for each independent simulation. For each combination of the four SD transitions and the three epistasis types, we ran 1000 independent simulations.

### 2.4 Data analysis

Model simulations, data analyses and data visualization were performed in R (v. 4.0.2; R Development Core Team, 2020) and RStudio (v. 1.2.5033; RStudio Team, 2020) using the ‘cowplot’ (Wilke, 2019), ‘mgcv’ (Wood, 2017), ‘viridis’ (Garnier, 2018) and ‘tidyverse’ (Wickham et al., 2019) packages. To interpolate between sampled parameter values, we fitted generalized additive models (GAMs) with binomial distribution and logit link to the rounded frequency (i.e., 0 or 1) of focal SD genes on either the paternally inherited (A in Y→A transitions) or maternally inherited (W in Y→W transitions) allele. In our simulations, allele frequencies of SD genes typically evolve to frequencies that are very close to 0 and 1, but may nonetheless not fully reach either value. This can result in a failure to fit a binomially distributed GAM; to prevent this issue, we round these allele frequencies. We used a full tensor smooth spline between the epistasis effect size ε and the selective effect parameters of the SA loci involved in the SD transition as predictor variables (Y→A: SA^Y^ and SA^A^; Y→W: SA^Y^ and SA^W^). In fitting the GAMs, we assumed a level‐specific trend and smoothness for each combination of epistasis type and the SA locus involved in epistasis. In addition, we fit separate GAMs for simulations where ε=0 to confirm that the outcome of SD transitions is unaffected by the types of epistasis when the effect of epistasis is zero; this analysis is performed separately as GAMs fail to distinguish between the qualitative difference between ε=0 and ε≠0. The GAM configurations used here correspond to a model I configuration as defined in Pedersen et al. (2019). Thin plate regression splines with extra shrinkage were used as base functions.

## 3 RESULTS

In our analysis, we focussed on the fitness effects of the SA loci linked to the ancestral and novel SD gene, as well as the effect size of epistasis on whether or not the new SD gene could invade or not. We additionally varied the type of SD transition (male heterogamety to male heterogamety (Y→A) or male heterogamety to female heterogamety (Y→W)), the type of epistatic interactions (coadaptation, dominance and overdominance), and which SA locus engaged in epistatic interactions. We find that the SA effects of the linked loci remain a key determinant of whether or not SD transitions may take place as described by Van Doorn and Kirkpatrick (2007, 2010). However, epistatic interactions of different types affect the range of parameter values for which transitions take place. We find that the parameter range resulting in an SD transition is differently affected depending on (1) the type of SD transition, (2) the type of epistasis and (3) the gene which interacts with EPI.

**FIGURE 2:**
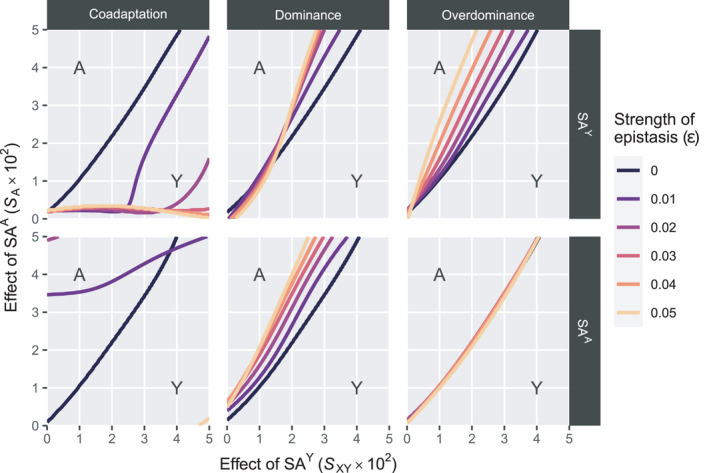
Maintenance of Y male heterogamety versus transition to A male heterogamety in Y→A transitions. Y may be maintained as the sex‐determining locus depending on the strength of SA effects associated with SA^Y^ (horizontal axis) and SA^A^ (vertical axis) as well as the effect of epistasis (differently coloured lines). Lines indicate boundaries for the maintenance of Y, with Y generally being maintained when parameter values are below the boundary line and A invading when they are above the line (see indications in the plots). An exception applies for the coadaptation epistasis scenario involving SA^A^; when ε=0.05, A invades below the boundary line rather than above it (see also Figure S1). Horizontal bars indicate different epistasis types, whereas vertical bars indicate the SA locus involved in epistasis with EPI

For the Y→A scenarios, we find that interactions between SA^Y^ and EPI tend to have a stabilizing effect on the Y allele as the male determiner for dominance and overdominance epistasis (Figure 2). More specifically, the minimal sexually antagonistic fitness effect of SA^A^ that results in an SD transition from Y to A is higher when SA^Y^ interacts with EPI. The stabilizing effect is more pronounced when the effect of epistasis ε is higher; that is, epistasis has a stronger effect. This effect however does not apply for overdominance epistasis involving SA^A^, where we instead observe that the scope for turnover is virtually unaffected (Figure 2, lower right panel). In contrast to the stabilizing effect of epistasis for dominance and overdominance epistasis, we find that for coadaptation epistasis, the effect of epistasis tends to be destabilizing except for when SA^A^ is involved in epistasis (Figure 2, Figure S1). When SA^Y^ interacts with EPI, we find that A can invade for a large range of parameter values except for when epistasis is weak. Similarly, when SA^A^ interacts with EPI, we find that A fails to invade and instead Y is maintained.

**FIGURE 3:**
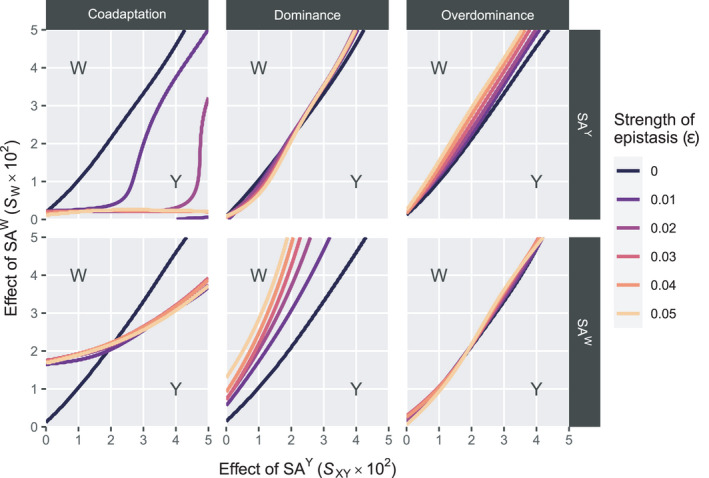
Maintenance of Y male heterogamety versus transition to W female heterogamety in Y→W transitions. Y may be maintained as the sex‐determining locus depending on the strength of SA effects associated with SA^Y^ (horizontal axis) and SA^W^ (vertical axis) as well as the effect of epistasis (differently coloured lines). Lines indicate lower boundaries for the invasion of W, with W being unable to invade and therefore Y being maintained as the sex‐determining gene when parameter values are below the boundary line and W invading and Y being fixed when they are above the line (see indications in the plots). An exception applies for the coadaptation epistasis scenario involving SA^Y^; when ε=0.01, W invades above the top‐left boundary line and below the bottom‐right boundary line (see also Figure S2). Horizontal bars indicate different epistasis types, whereas vertical bars indicate the SA locus involved in epistasis with EPI

In the Y→W transitions, we find that the effects of epistasis on the scope for turnover are comparable with those for Y→A transitions (Figure 3). Some differences do however exist; first, the effects of epistasis are much weaker for overdominance and dominance epistasis when SA^Y^ is involved. We find that overdominance epistasis involving SA^W^ has virtually no effect on the invasive capacity of W, which is similar to the case in Y→A transitions where overdominance epistasis involving SA^A^ does not affect the scope for turnover. Taken together, overdominance epistasis involving the SA locus linked to the novel SD gene appears to have no effect on the conditions which permit this new SD gene to invade. For coadaptation epistasis, we again find that when SA^Y^ is involved, this tends to promote turnover to W (Figure 3, Figure S2). When SA^W^ is involved, the dynamics are slightly more complicated; when the sexually antagonistic fitness effect of SA^Y^ is relatively weak, the effect of epistasis tends to favour its maintenance as the SD gene for higher values of W. However, as the selective effects associated with SA^Y^ are higher, the scope for turnover becomes larger. Interestingly, the strength of epistasis appears not to have a major effect on the scope for turnover; rather, it is only the presence/absence of epistasis that affects the outcome.

## 4 DISCUSSION

We investigated whether epistatic interactions can affect the stability of and explain turnovers in SD mechanisms. Our model builds on previous work by Van Doorn and Kirkpatrick (van Doorn & Kirkpatrick, 2007, 2010), who showed that SA selection can drive evolutionary transitions between SD mechanisms. Our model is an extension in that male fitness can be affected by an epistatic interaction between an SA locus on the ancestral or the novel pair of sex chromosomes and a neutral autosomal locus. We considered transitions between different male heterogamety systems and from male heterogamety to female heterogamety in combination with three different types of epistatic interactions. We furthermore varied the strength of epistasis and the SA loci involved, and whether the ancestral sex chromosome or the invading sex chromosome is involved in epistasis.

We found that epistasis can affect the scope for SD transitions, but the manner in which it does so depends on a variety of factors. For dominance and overdominance epistasis, epistasis tends to have either very little effect on the outcome of SD transitions (e.g., overdominance involving SA^A^ or SA^W^) or tends to promote maintenance of the ancestral sex chromosome pair. A possible explanation is that the allele frequencies of the SA locus on the ancestral sex chromosome pair have already diverged between the X‐ and Y‐chromosome. As the frequency of SA^Y^ increases on the Y‐chromosome, interactions between EPI and SA^Y^ occur more frequently than interactions between EPI and SA^A^ or SA^W^, who start out as autosomal SA genes and hence have a lower frequency in males. Effectively, under these conditions epistatic interactions are capable of enhancing stability of an ancestral SD system, but fail to enhance the invasive capacity of a new SD system; therefore, the effects of epistasis do not equally affect all SD genes. Instead, differentiation of the established sex chromosome pair leads to enrichment for alleles that engage in epistasis, thereby promoting its stability. Autosomal loci cannot become differentiated, so that they are not enriched for alleles involved in epistasis, and therefore, novel autosomal SD alleles do not experience the same benefit from epistatic interactions.

For coadaptation epistasis, the effects of epistasis tended to be destabilizing so that it facilitates turnover. In this scenario, doubly homozygous males (e.g., SA^Y^/SA^Y^; EPI/EPI or +/+; +/+ genotypes) experience a fitness benefit from epistasis. When the epistasis effect becomes sufficiently strong, it tends to favour transition to a state where the SA^Y^ and EPI alleles are both fixed or lost, depleting the genetic variance at this locus so that the X‐ and Y‐chromosome are no longer differentiated. This negates the possibility for SA selection, which would normally help maintain the sex chromosome pair, and instead opens up the scope for new SD genes to invade as predicted by the basic models by Van Doorn and Kirkpatrick (2007, 2010) on which our models were based. In their model, the ancestral SD system is maintained by selection favouring a pair of genetically differentiated haplotypes on one linkage group, for example, an X‐chromosome and a Y‐chromosome that are enriched respectively for female‐ and male‐beneficial alleles. Invasion of the novel sex chromosome system occurs when selection favours the evolution of differentiated haplotypes on this linkage group more strongly than on the ancestral sex chromosomes. In our model, fixation or loss of SA^Y^ prevents selection from favouring distinct female‐beneficial X‐chromosomes and male‐beneficial Y‐chromosomes, so that this mechanism only applies to the linkage group carrying the novel SD gene. The disruptive effect of coadaptation epistasis on the sex chromosomes involved might have occurred because we assumed that this type of epistasis only occurs in double homozygotes. Instead, Y‐chromosomal loci may be considered to be hemizygous so that coadaptation epistasis could occur in SA^Y^; EPI/EPI or +; +/+ genotypes. This could have a stabilizing effect as SA genetic variation between the X‐ and Y‐chromosome may persist while epistasis strengthens the benefit of bearing the Y‐chromosome. As coadaptation epistasis in our model favours fixation of the SA locus involved for either allele, it nullifies the ability for SA selection to favour the spread or maintenance of the linked SD locus. This results in the destabilization of the existing SD system or the inability of novel SD genes to invade.

The effects of epistasis provide another explanation for the apparent stability of some sex chromosome systems such as those of most mammals. Here, the sex chromosome system may not be stable solely due to the characteristics of a given SD gene (e.g., being insensitive to becoming regulated by a newly evolved upstream SD gene), but rather because of genetic differentiation of the region linked to the SD gene. This includes for example male‐essential genes on the Y‐chromosome that prevent its loss (e.g., as in the case of the Y→A transitions) or the decayed nature of older Y‐chromosomes preventing fixation of Y, as homozygous YY individuals experience severe fitness costs (e.g., as in the case of Y→W transitions) (Bull & Charnov, 1977; Graves, 2006; van Doorn, 2014). Both of these effects however mostly apply to older sex chromosome pairs that have already persisted for extended periods of time. In contrast, the stabilizing effect of epistasis as reported here can apply from the very onset of sex chromosome evolution, as we show that a single locus involved in epistasis may already affect the stability of the sex chromosome system. This means that the stabilizing effect of epistatic interactions may occur on relatively undifferentiated sex chromosomes. Although these effects are less substantial, we find they can be sufficient to prevent early displacement of an SD gene once established (although a sufficiently strong selective pressure on the new SD gene may still enable a transition). Over time, other factors such as acquisition of male‐essential genes or recessive deleterious mutations may then further enhance the stability of ancestral sex chromosomes so that these can persist over extended periods of time.

As a caveat to the above, it must be noted that the effects of epistasis appear to depend on the type of SD transition considered, with the effects of epistasis being more pronounced in Y→A transitions as compared to Y→W transitions. A possible explanation is that in the latter, Y is fixed rather than lost. If the Y‐bearing chromosome has become enriched for SA^Y^ alleles (as described in Jordan & Charlesworth, 2012; Rice, 1987), the frequency of male‐beneficial epistatic interactions does not decrease directly as W spreads in the population. This instead only drops later as the Y‐bearing chromosome is no longer male‐restricted, and therefore, the frequency of SA^Y^ on this chromosome decreases as well. Even then, the frequency of SA^Y^ in this new ‘quasi‐autosomal’ state may still be higher than the frequency of SA^Y^ on the ancestral X‐chromosome (i.e., the non‐Y‐bearing chromosome that existed prior to the spread of W and fixation of Y), which instead had been enriched for the female‐beneficial non‐focal allele at the SA^Y^ locus. In Y→A transitions, A‐bearing males must also bear two such ‘X‐chromosomes’ which severely reduces their odds of experiencing the benefits of epistasis. This poses an additional burden to the invasion of A that does not apply to invasion of W.

We focussed here specifically on a model involving SA loci, but other mechanisms capable of driving SD transitions may likewise be modulated by the effects of epistatic interactions (e.g., meiotic drive (Kozielska et al., 2010)). The benefit of Y‐chromosomal differentiation with regard to SA loci, resulting in an increased frequency of epistasis, may apply more broadly to other genes as well, with the only requirement being that the Y‐chromosome becomes enriched for an allele that engages in epistatic interactions. Examples of this include the evolution of Y‐chromosomal regulating genes such as those regulating the expression of autosomal SA genes (Ågren et al., 2019). Y‐chromosomes (or W‐chromosome in ZW systems) of several species have essential regulatory functions (Lahn & Page, 1997; Wright et al., 2014), as evident from their gene content and the impact of Y‐chromosomal genetic variation in a variety of species (e.g., Bellott et al., 2014; Lemos et al., 2008). Therefore, given that Y‐autosome interactions are prevalent in many species, the effects of such interactions on SD transitions may likewise apply in many organisms.

In this study, we have explored the effect of epistatic interactions between a sex chromosome (either ancestral or novel) and an autosome on the scope of turnover from an ancestral to a novel sex chromosome system. Our results demonstrate that such interactions can confer additional stability to an ancestral sex chromosome system for some types of epistatic interactions, whereas other interactions can reduce the stability of the ancestral sex chromosomes. The capacity for sex chromosomes to become genetically differentiated relative to autosomes here enables epistatic effects to become more prevalent and/or pronounced, thereby resulting in increased stability of established systems. When a novel SD gene evolves on an autosome, no such genetic differentiation has occurred and therefore epistasis benefits the spread of novel SD genes to a lesser extent. The effect of epistasis on transitions in SD is further largely dependent on the type of sex chromosome transition considered and the strength of epistasis. In conclusion, the stability of a sex chromosome pair does not depend solely on its own characteristics, but instead should be considered as part of an interactive network with the remainder of the genome.

## CONFLICT OF INTEREST

The authors declare no conflicts of interest.

## AUTHOR CONTRIBUTIONS

MAS, LWB and IP conceived and designed the study; MAS collected and analysed the data; MAS drafted the initial version of the manuscript; MAS, LWB and IP contributed to later versions of the manuscript.

### PEER REVIEW

The peer review history for this article is available at https://publons.com/publon/10.1111/jeb.13939.

### OPEN RESEARCH BADGES

This article has been awarded Open Data, Open Materials Badges. All materials and data are publicly accessible via the Open Science Framework at https://doi.org/10.5061/dryad.9s4mw6mhf, https://zenodo.org/record/5513638#.YUSDhX1cKHs.

## Supporting information

Fig S1

Fig S2

Supplementary Material

Supplementary Material

## Data Availability

All source code for the model and all analysis scripts are available through GitHub (https://github.com/MartijnSchenkel/EpistasisSexDetermination). Primary data generated using the source code are deposited on Dryad (https://doi.org/10.5061/dryad.9s4mw6mhf).

## 1 Detailed description of model

### 1.1 Model initialization

To initialize the model, we first determine the frequency of different haplotypes at each linkage group based on a series of starting frequencies for the focal allele 1 (as described in the main text) on the maternally inherited (first) and the paternally inherited (second) copy, which are used to calculate the frequencies of the possible haplotypes on each linkage group at the first and the second copy. For example, a 11 haplotype (which indicates a focal SD allele linked to a focal SA allele) on the maternal copy of linkage group XY is given by $P\left(Y_{1}^{M}\right)\;\times P\left({\mathrm{SA}}_{1}^{Y}\right)$. For linkage group III, which only carries the EPI locus, the haplotype frequencies are given simply by the allele frequencies of alleles 0 and 1. We define an array $F_{i,j,k}$ which contains the frequencies of the different haplotypes linkage groups XY, I^A^ and II^W^, where i denotes linkage group (1 = XY; 2 = I^A^; 3 = II^W^), j denotes haplotype (1 = 00; 2 = 01; 3 = 10; 4 = 11), and k denotes the allele copy (1 = maternally inherited allele; 2 = paternally inherited allele). For linkage group III, we similarly define an array $F_{i,j}^{\mathrm{EPI}}$ where i and j indicate which allele is present on respectively the maternal and the paternal copy (for both i and j, 1 = allele 0; 2 = allele 1).

Based on $F_{i,j,k}$ and $F_{i,j}^{\mathrm{EPI}}$, we can define our initial population, which is given by an array P with dimensions H×H, where H represents a 4 × 4 × 4 × 2 array where each element represents a particular combination of haplotypes. Each element p in P represents a particular genotype, and the value of element p gives its frequency. The frequency of each genotype p in $P_{i,j,k,l,m,n,o,p}$ (hereafter we use the subscript i to indicate an array with dimensions i through p) is given by $F_{1,1,i}\;\times \;F_{2,1,j}\;\times \;F_{3,1,k}\;\times \;F_{1,2,m}\times \;F_{2,2,n}\;\times \;F_{3,2,o}\;\times \;F_{l,p}^{\mathrm{EPI}}$, where i and m indicate the haplotype on XY at the maternally and paternally inherited copies respectively, and similarly j and n, and k and o, for linkage groups I^A^ and II^W^; l and p indicate the genotype at the maternal and paternal copies of the EPI locus. Similarly, we define an array $N_{h,i}$ which counts the number of focal alleles 1 at locus h (1 = Y, 2 = SA^Y^, 3 = A, 4 = SA^A^, 5 = W, 6 = SA^W^, 7 = EPI) for genotype p in P; values in N can be 0 (homozygous 0/0), 1 (heterozygous 1/0 or 0/1) or 2 (homozygous 1/1).

Because we randomly select the parameter values for the SA loci and the epistasis effect, the population upon initiation does not conform to a population at equilibrium. We therefore incorporate 10,000 generations of burn in during which allele frequencies can reach equilibrium prior to introducing new SD genes via mutation. This mutation procedure is described under ‘Gametogenesis and reproduction’.

### 1.2 Sex determination

Sex determination takes place based on the number of focal alleles 1 at loci Y, A and W in each genotype (see Table S1). To do so, we define two binary arrays $S^{M}$ and$S^{F}$, which describes for each genotype in whether that genotype is male ($S^{M}\;=\;1$ and$S^{F}\;=\;0$) or female ($S^{M}\;=\;0$ and$S^{F}\;=\;1$); we use superscripts F and M to identify arrays containing female‐ and male‐specific components of the model throughout. A genotype in $P_{i}$ is male when (1) $N_{5,i}$ = 0 and (2) $N_{1,i}$ > 0 and/or $N_{3,i}$ > 0. The frequencies of different genotypes in males ($P^{M}$) are given by the entrywise product of $P\;\circ \;S^{M}$, and the frequencies in females ($P^{F}$) are given by$P\;\circ \;S^{F}$. To keep track of the frequencies of the focal alleles per sex, the frequency of the focal allele at locus h in males is given by the inner product$P^{M}\cdot N_{hi}$, whereas those in females are given by$P^{F}\cdot N_{hi}$.

### 1.3 Sex‐specific fitness effects and epistasis

Fitness is determined by sex and the genotypes at loci SA^Y^, SA^A^ and SA^W^ and additionally by epistatic effects. For simplicity, we calculate for each genotype two fitness scores which assume that the genotype is respectively male ($W^{M}$) or female ($W^{F}$). For each SA locus, we can define a dominance parameter $h_{j}^{i}$ and fitness effect parameter $s_{j}^{i}$, where i indicates the linkage group (1 = XY; 2 = I^A^; 3 = II^W^) and j indicates sex (1 = male, 2 = female). We then define six fitness vectors: $w_{\mathrm{XY}}^{M}$ and $w_{\mathrm{XY}}^{F}$ (for fitness effects of SA^Y^ in males and females respectively), $w_{A}^{M}$ and $w_{A}^{F}$ (same, but for SA^A^), and $w_{W}^{M}$ and $w_{W}^{F}$ (for SA^F^). The fitness scores in $w_{\mathrm{XY}}^{M}$ are given by $\left\{1,\;1+h_{\mathrm{XY}}^{M}\times s_{\mathrm{XY}}^{M},\;1+s_{\mathrm{XY}}^{M}\right\}$, and those in $w_{\mathrm{XY}}^{F}$ are given by $\left\{1,\;1+h_{\mathrm{XY}}^{F}\times s_{\mathrm{XY}}^{F},\;1+s_{\mathrm{XY}}^{F}\right\}$. Similarly, the fitness scores in $w_{A}^{M}$ and $w_{A}^{F}$ are calculated using $h_{A}^{j}$ and $s_{A}^{j}$, and those in $w_{W}^{M}$ and $w_{W}^{F}$ are calculated using $h_{W}^{j}$ and $s_{W}^{j}$. Using these fitness scores, we can calculate the fitness scores of all genotype frequencies. Let a indicate the number of focal alleles at the SA^Y^ locus of genotype p in P, which is given by $N_{2}$, and similarly b (given by $N_{4}$) and c (given by $N_{6}$) be the number of focal alleles at loci SA^A^ and SA^W^, respectively. The male fitness score of genotype p in P ($W^{M}$) is then given by ${w_{\mathrm{XY}}^{M}}_{a+1}\times \;{w_{A}^{M}}_{b+1}\times \;{w_{W}^{M}}_{c+1}$ and similarly its female fitness score ${(W}^{F})$ is given by ${w_{\mathrm{XY}}^{F}}_{a+1}\times \;{w_{A}^{F}}_{b+1}\times \;{w_{W}^{F}}_{c+1}$ (note that the +1 here is necessary to ensure that the proper index is used, i.e., a male with 0/0 genotype at SA^Y^ has 0 focal alleles at this locus, but its fitness score for this locus corresponds to ${w_{\mathrm{XY}}^{F}}_{1}$ = 1). The genotype frequencies in females after selection ($G^{F}$) are given by $P^{F}\circ W^{F}$.

Because our model incorporates epistatic effects on fitness in males, the genotype frequencies in males after selection ($G^{M}$) are given by $P^{M}\circ W^{M}\circ E$, where E indicates the epistasis fitness component. Epistatic effects occur as a result of an interaction between an SA locus and the EPI locus. The epistasis fitness component is given by E=1+σε, in which ε denotes the epistasis effect size (which we vary between 0 and 0.05 for different simulations) and σ modulates the effects of epistasis. We use three different epistasis scenarios which all assume epistatic effects occur for different genotype combinations (see also Table 3):

1. Dominance: $\sigma_{i}\;=\;1$ if $N_{k,i}\;\geq 1$ and $N_{7,i}\;\geq 1$; $\sigma_{i}\;=\;0$ for all other genotype combinations.
2. Overdominance: $\sigma_{i}\;=\;1$ if $N_{k,i}\;=1$ and $N_{7,i}\;=1$; $\sigma_{i}\;=\;0$ for all other combinations.
3. Coadaptation: $\sigma_{i}\;=\;1$ if $N_{k,i}\;=0$ and $N_{7,i}\;=0,$ or $N_{k,i}\;=2$ and $N_{7,i}\;=2$; $\sigma_{i}\;=\;0$ for all other genotype combinations.

Here, k indicates which SA locus is involved in epistasis (k=2 for SA^Y^, k=4 for SA^A^ and k=6 for SA^F^).

### 1.4 Gametogenesis and reproduction

Reproduction takes place by gametogenesis in males and females to yield pools of sperm and oocytes, respectively. To calculate the frequencies of the haplotypes amongst the sperm and oocytes, we first define an array $U_{i,j,k,l}$, in which i denotes linkage group (1 = XY; 2 = I^A^; 3 = II^W^), jand kdenote haplotype on the maternally inherited and paternally inherited chromosome copies, respectively, and ldenotes the haplotype to be sampled (for j, kand l: 1 = 00; 2 = 01; 3 = 10; 4 = 11), such that element $u_{i,j,k,l}$gives the probability of sampling a haplotype lfrom a diploid genotype consisting of haplotypes jand kon linkage group i. In defining U, we also account for recombination which may yield novel haplotypes; for example, a genotype 00/11 can yield 01 and 10 haplotypes if recombination occurs (see Table S2). Similarly, we define an array $V_{i,j,k,l}$, where jdenotes the haplotype at the paternally inherited allele at the EPI locus, kdenotes that of the maternally inherited allele, and idenotes the type of allele to be sampled (for i, jand kalike, 1 = the non‐focal allele 0, 2 = the focal allele 1), such that element $v_{i,j,k,l}$gives the likelihood of sampling an allele of type ifrom a diploid genotype of jand k. Uand Vcan be used to construct an array T, which is defined as $T_{i,j,k,l,m,n,o,p,q,r,s,t}=U_{1,i,m,q}\times U_{2,j,n,r}\times U_{3,k,o,s}\times V_{l,p,t}$(note that the order of subscripts in Uand Vhere deviates from that used above). The matrix product of $G^{F}T$yields an array $H^{F}$containing the frequencies of each haplotype among the oocytes; similarly, the matrix product of $G^{M}T$gives an array $H^{M}$which describes the haplotype frequencies among sperm. Genotypes are formed by fusion of sperm and oocytes, with genotype frequency being given by the product of their respective frequencies, such that the frequencies of genotypes in the next generation are given by the Kronecker product $H^{F}⊗H^{M}$, yielding an array Q. Qhas identical dimensions to Pand effectively represents the offspring produced by this ancestral population P. Therefore, we can update P=Qto represent moving forward one generation in our model. All simulations are carried out for 200,000 generations, during which we track the frequency of the focal allele 1 at each locus.

We introduce either A and W by mutations in gametes by manipulation of the $H^{F}$ and $H^{M}$ arrays. To introduce A into the population, in both the $H^{F}$ and $H^{M}$ arrays, we redefine $H_{i3kl}=H_{i1kl}\times \mu_{A}$ and $H_{i4kl}=H_{i2kl}\times \mu_{A}$ to convert a proportion $\mu_{A}$ of 00 and 01 gametes into 10 and 11 gametes; we also redefine $H_{i1kl}=H_{i1kl}\times \left(1‐\mu_{A}\right)$ and $H_{i2kl}=H_{i2kl}\times {(1‐\mu }_{A})$. Similarly for introducing W, we redefine $H_{ij3l}=H_{ij1l}\times \mu_{W}$ and $H_{ij4l}=H_{ij2l}\times \mu_{W}$ to the same effect, and redefine $H_{i1kl}=H_{i1kl}\times \left(1‐\mu_{W}\right)$ and $H_{i2kl}=H_{i2kl}\times {(1‐\mu }_{W})$. In Y→A transitions, we introduce A at a frequency $\mu_{A}=10^{‐4}$ whereas in Y→W transitions, we introduce W at an identical frequency $\mu_{W}=10^{‐4}$. A and W are introduced after 10,000 generations of burn in during which the initial population has been allowed to evolve to reach an equilibrium as dependent on the selective effect parameter values for the various SA genes and the epistatic effect.

### 1.5 Parameter value selection

In this manuscript, we focus on the fitness effects of the SA genes and the effect of epistasis primarily, and therefore do not vary some other aspects described here such as the recombination rates between the SD and SA genes or the dominances of the SA genes in males and females. The parameter range for the SA effects is taken to reflect a fitness effect between 0% and 5% in SA/SA homozygotes (where SA reflects the focal allele for a given SA locus) relative to +/+ homozygotes. To do so, we sampled the fitness effects for each SA locus from a uniform distribution with range (0, 0.5); the epistasis effect size ε is sampled from an identical uniform distribution. Fitness effects are identical in males and females but with inverse sign, that is, a fitness effect s in males is associated with a fitness effect ‐s in females and vice versa. In line with this, we assume a low rate of recombination (1%) which generally results in a case of strong linkage as defined by van Doorn and Kirkpatrick (2007). Dominances for the fitness effects of SA alleles are set to 0.6 in the sex in which they have a beneficial effect (males for SA^Y^ and SA^A^, females for SA^W^) and 0.4 in the sex in which they have a deleterious effect. This configuration for recombination rate and dominance parameters should promote the maintenance of SA polymorphism for a large range of fitness effects (Jordan & Charlesworth, 2012). For both Y→A and Y→W transitions, we only consider fitness effects of SA alleles for those loci that are linked to one of the potential SD genes, for example, in Y→A transitions we include a fitness effect for SA^Y^ and SA^A^, but not for SA^W^ whose fitness effect is instead taken to be zero.
