# Supplementary figures and images for: Epistatic interactions between sex chromosomes and autosomes can affect the stability of sex determination systems
Source: J Evol Biol. 2021 Oct 1;34(11):1666–77. doi: 10.1111/jeb.13939 (PMC9291586; doi:10.1111/jeb.13939)

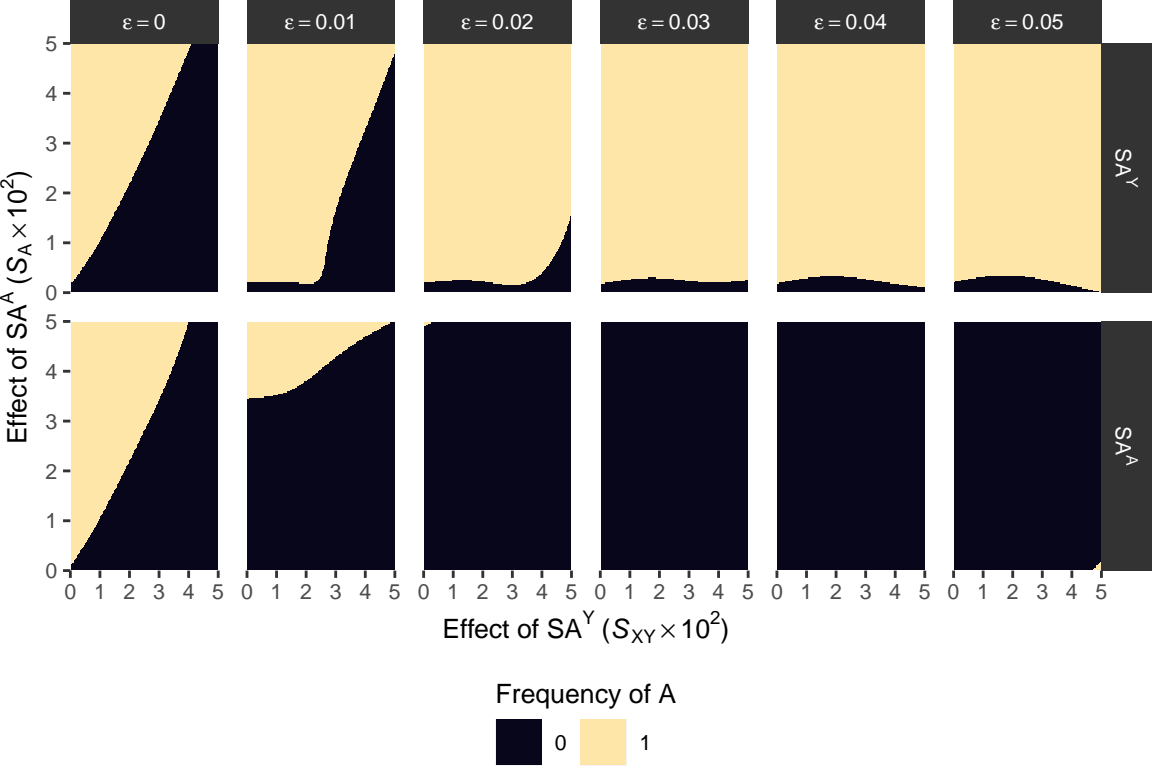

Supplement: Supplementary file 1 — Fig S1 [file JEB-34-1666-s003.pdf]

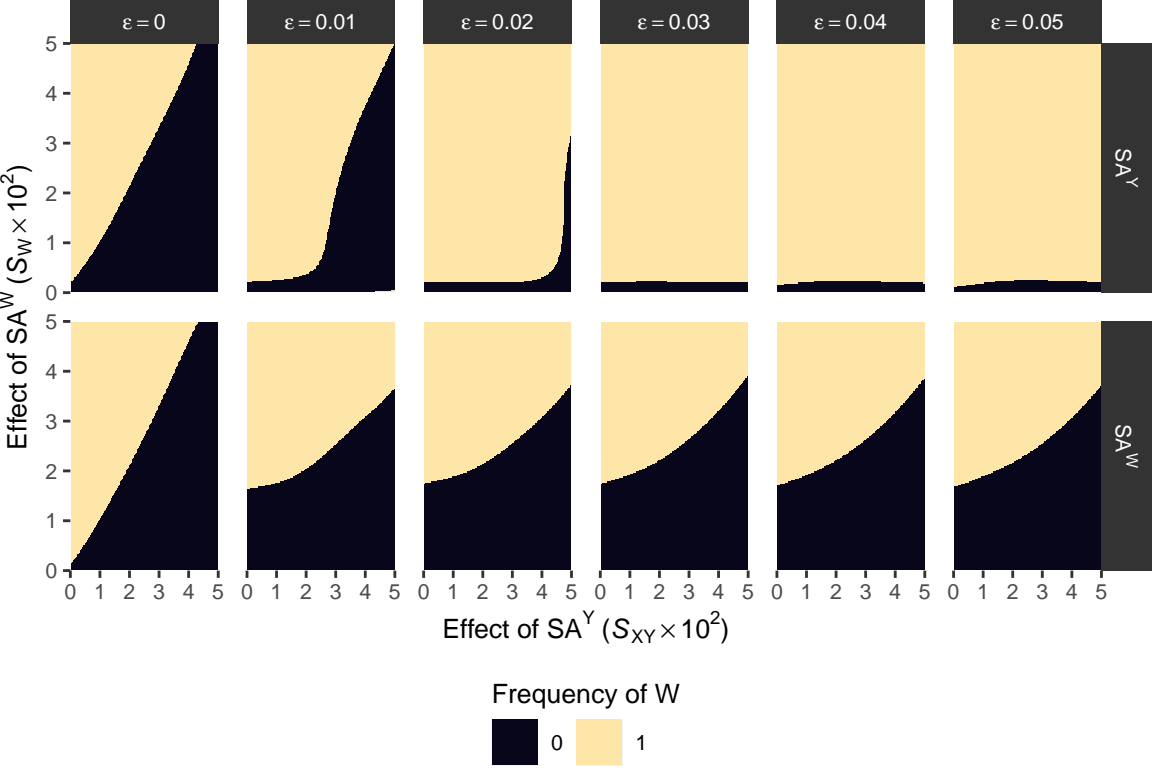

Supplement: Supplementary file 2 — Fig S2 [file JEB-34-1666-s004.pdf]
