# Supplementary material for: Epistatic interactions between sex chromosomes and autosomes can affect the stability of sex determination systems
Source: J Evol Biol. 2021 Oct 1;34(11):1666–77. doi: 10.1111/jeb.13939 (PMC9291586; doi:10.1111/jeb.13939)
Supplement: Supplementary file 3 — Supplementary Material [file JEB-34-1666-s001.docx]

# Supplementary Tables

**Supplementary Table S1.** Default parameter values used for different SD transition scenarios.

| **Variable** | **Y→A** | **Y→W** | **Description** |
| --- | --- | --- | --- |
| $r_{\mathrm{XY}}$ | 0.01 | 0.01 | Recombination rate between Y and SA^Y^ |
| $r_{A}$ | 0.01 | 0 | Recombination rate between A and SA^A^ |
| $r_{W}$ | 0 | 0.01 | Recombination rate between W and SA^W^ |
| $h_{\mathrm{XY}}^{M}$ | 0.6 | 0.6 | Dominance of SA^Y^ in males |
| $h_{A}^{M}$ | 0.6 | 0 | Dominance of SA^A^ in males |
| $h_{W}^{M}$ | 0 | 0.4 | Dominance of SA^W^ in males |
| $h_{\mathrm{XY}}^{F}$ | 0.4 | 0.4 | Dominance of SA^Y^ in females |
| $h_{A}^{F}$ | 0.4 | 0 | Dominance of SA^A^ in females |
| $h_{W}^{F}$ | 0 | 0.6 | Dominance of SA^W^ in females |

| **Supplementary Table S2.** Proportion of haplotypes produced by diploid genotypes. | | | | | |
| --- | --- | --- | --- | --- | --- |
| **Maternal copy** | **Paternal copy** | **P(00)** | **P(01)** | **P(10)** | **P(11)** |
| **00** | **00** | $1$ | $0$ | $0$ | $0$ |
|  | **01** | $0.5$ | $0.5$ | $0$ | $0$ |
|  | **10** | $0.5$ | $0$ | $0.5$ | $0$ |
|  | **11** | $(1-r_{n})/2$ | $(r_{n})/2$ | $(r_{n})/2$ | $(1-r_{n})/2$ |
| **01** | **00** | $0.5$ | $0.5$ | $0$ | $0$ |
|  | **01** | $0$ | $1$ | $0$ | $0$ |
|  | **10** | $(r_{n})/2$ | $(1-r_{n})/2$ | $(1-r_{n})/2$ | $(r^{n})/2$ |
|  | **11** | $0$ | $0.5$ | $0$ | $0.5$ |
| **10** | **00** | $0.5$ | $0$ | $0.5$ | $0$ |
|  | **01** | $(r_{n})/2$ | $(1-r_{n})/2$ | $(1-r_{n})/2$ | $(r_{n})/2$ |
|  | **10** | $0$ | $0$ | $1$ | $0$ |
|  | **11** | $0$ | $0$ | $0.5$ | $0.5$ |
| **11** | **00** | $(1-r_{n})/2$ | $(r_{n})/2$ | $(r_{n})/2$ | $(1-r_{n})/2$ |
|  | **01** | $0$ | $0.5$ | $0$ | $0.5$ |
|  | **10** | $0$ | $0$ | $0.5$ | $0.5$ |
|  | **11** | $0$ | $0$ | $0$ | $1$ |

$r_{n}$ indicates the recombination rate on linkage group *n* (XY, I^A^, or II^W^).

# Supplementary Figures


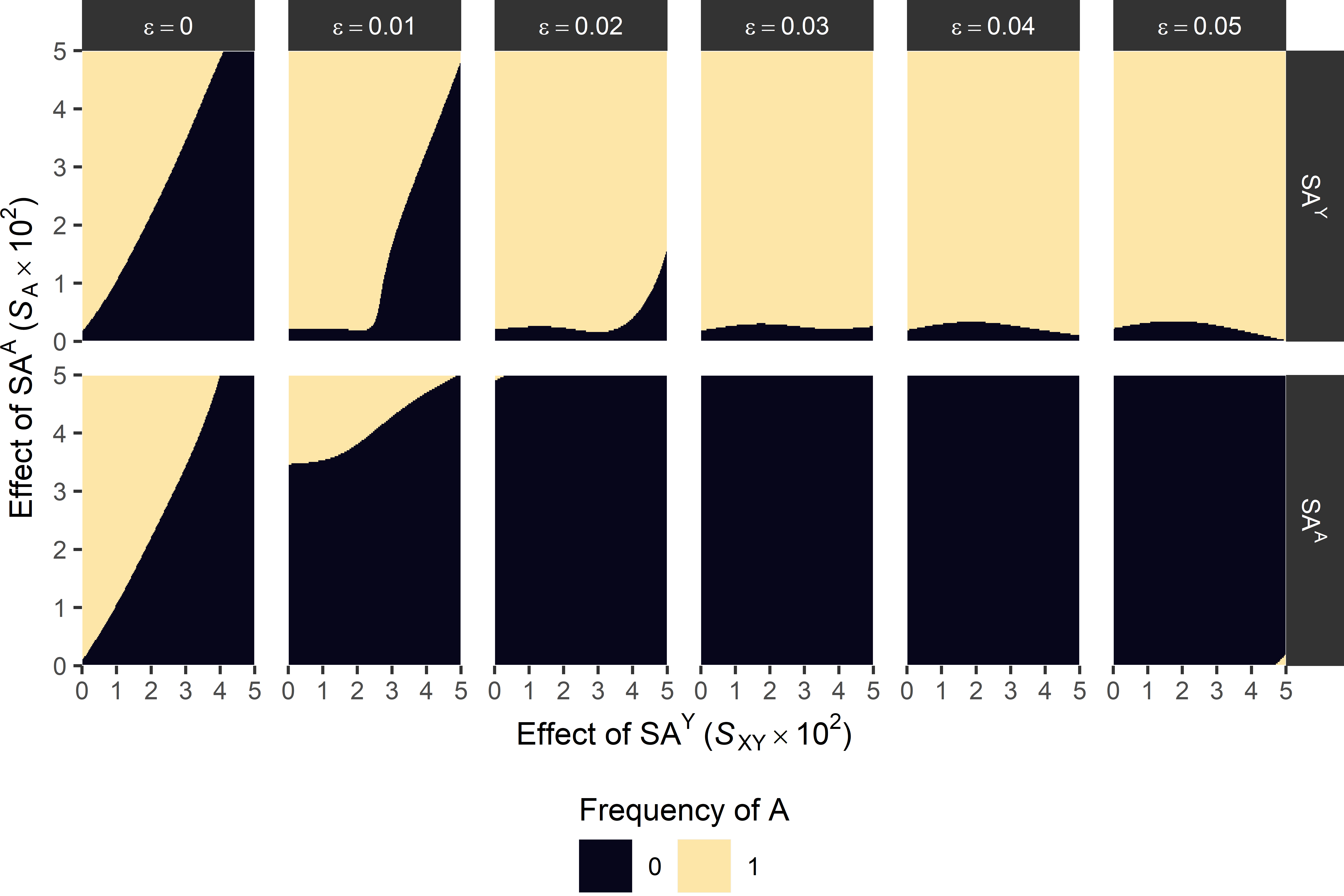


**Supplementary Figure 1:** Invasion of A in Y→A transitions with coadaptation epistasis. Shown here are the predicted allele frequencies of the A allele on the paternally-inherited chromosome in males at equilibrium. Predicted frequencies were generated using a GAM with fitted to the observed allele frequencies, with a full tensor smooth between the effects of SA^Y^, SA^A^, and epistasis effect size (for details see main text). Horizontal bars indicate different epistasis effect sizes ($\varepsilon$), whereas vertical bars indicate the SA locus involved in epistasis. Note that when SA^A^ interacts with EPI and $\varepsilon=0.05$, A invades below the boundary line depicted in Figure 2 rather than above it (here indicated in the bottom right corner of the right-most panel on the bottom row).


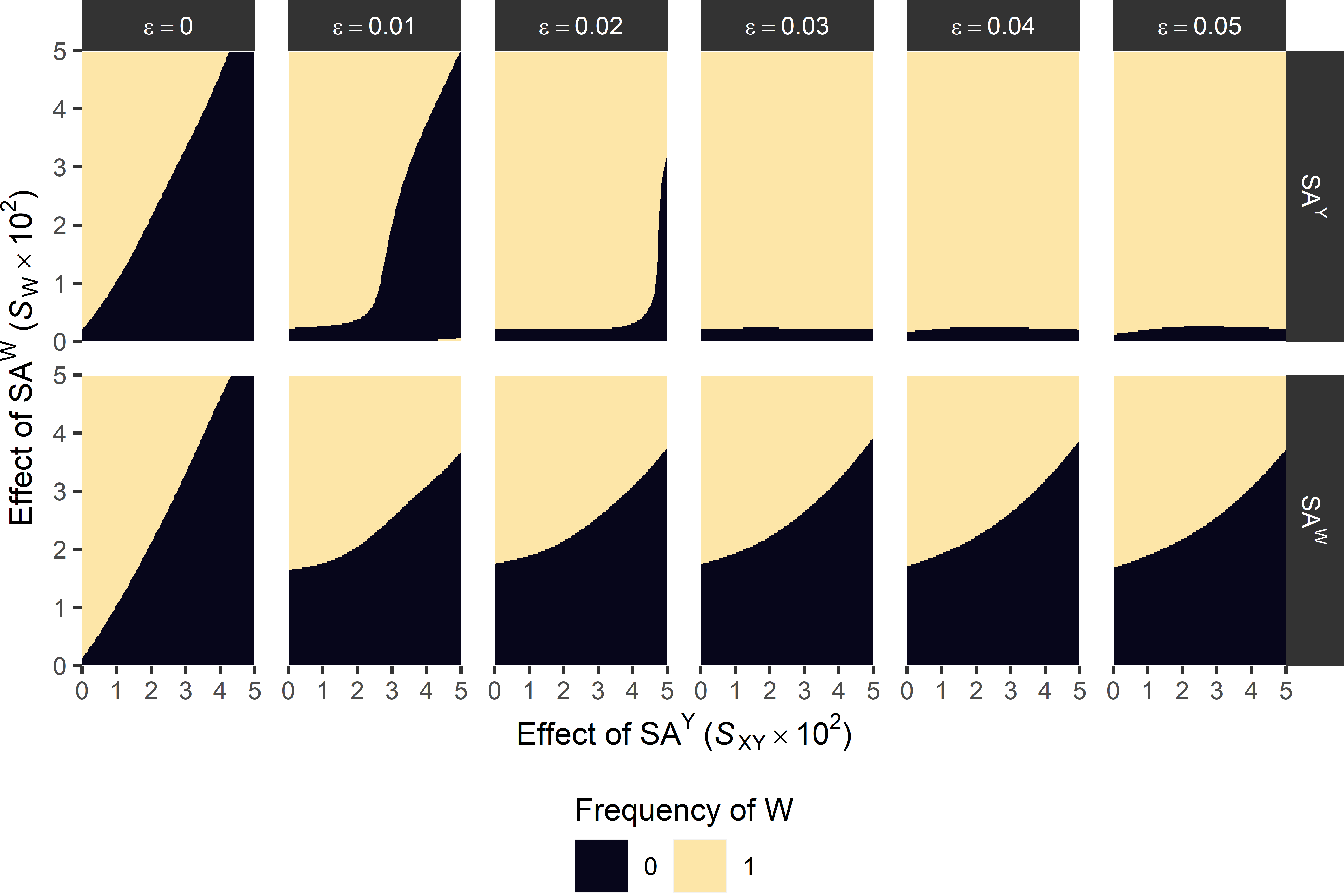


**Supplementary Figure 2:** Invasion of W in Y→W transitions with coadaptation epistasis. Shown here are the predicted allele frequencies of the W allele on the maternally-inherited chromosome in females at equilibrium. Predicted frequencies were generated using a GAM with fitted to the observed allele frequencies, with a full tensor smooth between the effects of SA^Y^, SA^W^, and epistasis effect size (for details see main text). Horizontal bars indicate different epistasis effect sizes ($\varepsilon$), whereas vertical bars indicate the SA locus involved in epistasis. Note that when SA^Y^ interacts with EPI and $\varepsilon=0.01$, W invades above the top-left boundary line and below the bottom-right boundary line as depicted in Figure 3 (here indicated in the upper left corner and lower right corner of the second panel on the top row).
